# Extracellular vesicles in colorectal cancer: M2 polarization, bidirectional regulation of CAFs, metastasis, and treatment

**DOI:** 10.3389/fcell.2026.1980961

**Published:** 2026-10-12

**Authors:** Xiaoling Wang, Zhaohua Lai, Hanlu Chen, Mei Yang, Huapei Kang, Fangsheng Wang, Junyun Huang, Yuxin Deng

**Affiliations:** 1 Department of Laboratory Medicine, The First Affiliated Hospital of Gannan Medical University, Ganzhou, China; 2 College of Medical Technology, Gannan Medical University, Ganzhou, China; 3 The First School of Clinical Medicine, Gannan Medical University, Ganzhou, China; 4 Center for Clinical Laboratories, The Affiliated Hospital of Guizhou Medical University, Guiyang, China

**Keywords:** colorectal cancer, extracellular vesicles, macrophages, mesenchymal stem cell, tumor microenvironment

## Abstract

Extracellular vesicles (EVs), as key mediators of intercellular communication, play a critical regulatory role in the pathogenesis and treatment of colorectal cancer (CRC). By carrying diverse bioactive molecules—including miRNAs, lncRNAs, circRNAs, and DNA—EVs influence multiple components of the tumor microenvironment (TME), such as promoting macrophage M2 polarization, reducing T and B Cell activity and proliferation, enabling evasion of natural killer (NK) cell surveillance, and regulating bidirectional fibroblast interactions in CRC. Furthermore, CRC-derived EVs can promote CRC metastasis by stimulating angiogenesis in endothelial cells, facilitating epithelial-mesenchymal transition (EMT), and promoting the formation of pre-metastatic niches. Through an analysis of the role of EVs in the pathogenesis of CRC, numerous studies have found that EVs can augment the efficacy of immunotherapy. At the same time, both mesenchymal stem cell-derived EVs and engineered EVs (such as plant-derived and microorganism-derived EVs) can play significant roles in the treatment of CRC. However, most current research remains in the *in vitro* and *in vivo* trial stages. Due to difficulties in purification and engineering, as well as inherent heterogeneity, clinical translation remains challenging; consequently, no related products have yet been brought to market. Most clinical studies remain focused on diagnostic applications, while trials for therapeutic applications are still recruiting participants. Against this backdrop, we highlight the limitations and challenges in EVs research, with the aim of providing better research directions for the clinical translation of CRC treatments.

## 1 Introduction

According to the latest statistics from the International Agency for Research on Cancer, there were nearly 20 million new cancer cases worldwide in 2022. Among these new cases, colorectal cancer (CRC) accounted for 9.6% of new cases, ranking third in terms of new cancer cases. It is also the second leading cause of cancer-related deaths (Bray et al., 2024). The development of CRC is a complex, multi-factorial process that fundamentally depends on the dynamic remodeling of the tumor microenvironment (TME). The TME primarily comprises tumor cells, immune cells, fibroblasts, and stromal tissue, all of which influence tumor growth, metastasis, dissemination, and therapeutic response (Peng et al., 2024). Therefore, identifying the key mediators of intercellular communication within the TME is of critical importance for CRC diagnosis and therapy.

Extracellular vesicles (EVs) refer to particles that are released from cells, are delimited by a lipid bilayer, and cannot replicate on their own. EVs serve as important mediators of CRC progression (Welsh et al., 2024). EVs can be isolated in substantial quantities from various bodily fluids, including amniotic fluid, blood, cerebrospinal fluid, urine, saliva and breast milk, thus representing a promising non-invasive liquid biopsy tool for CRC diagnosis (Nieuwland et al., 2024). In addition, EVs participate in cell-to-cell communication between CRC cells and other cells by carrying DNA, RNA, lipids, proteins, and other molecules. Furthermore, EVs protect their cargo from enzymatic degradation, thereby playing a significant role in CRC pathogenesis (Lorite et al., 2024; Wang C. et al., 2025; Kamra et al., 2026). These characteristics of EVs make them suitable as delivery vehicles for CRC therapy; through certain biotechnological methods, EVs can be engineered into stable, targeted therapeutic delivery vehicles. Accumulating evidence indicates that EVs play a critical role in CRC pathogenesis. In terms of immune regulation, CRC-derived EVs promote the polarization of tumor-associated macrophages toward the M2 phenotype (Liang et al., 2024), inhibit CD8^+^ T-cell activity and proliferation (Shi et al., 2024), and contribute to the establishment of an immunosuppressive TME. Additionally, CRC-derived EVs impair dendritic cell maturation (Asadirad et al., 2022). Furthermore, CRC cell-derived EVs stimulate the transformation of fibroblasts into cancer-associated fibroblasts (CAFs), whereas CAF-derived EVs, in turn, act on CRC cells to promote their malignant phenotype (Yang et al., 2022; Zhou et al., 2026). EVs also promote angiogenesis and epithelial-mesenchymal transition (EMT) in CRC through multiple mechanisms (Pucci et al., 2023; Long et al., 2024). In summary, this review primarily (but not exclusively) covers studies published between 2022 and 2026. It also highlights the current status of clinical trials and the challenges associated with clinical translation, with the aim of providing a solid theoretical foundation for the clinical translation and application of EVs in CRC. We conducted a search in PubMed using the keywords “extracellular vesicles” and “colorectal cancer,” combined with keywords from various titles, and limited the search to the years 2022 through 2026.

## 2 The mechanism by which EVs regulate CRC pathogenesis

The bioactive molecules carried by EVs reshape the TME by modulating its diverse cellular and stromal components, thereby regulating the malignant phenotype of CRC. Furthermore, EVs participate in multiple metastatic processes of CRC, including epithelial-mesenchymal transition (EMT), angiogenesis, and pre-metastatic niche (PMN) formation. These processes are summarized in Figure 1.

**FIGURE 1:**
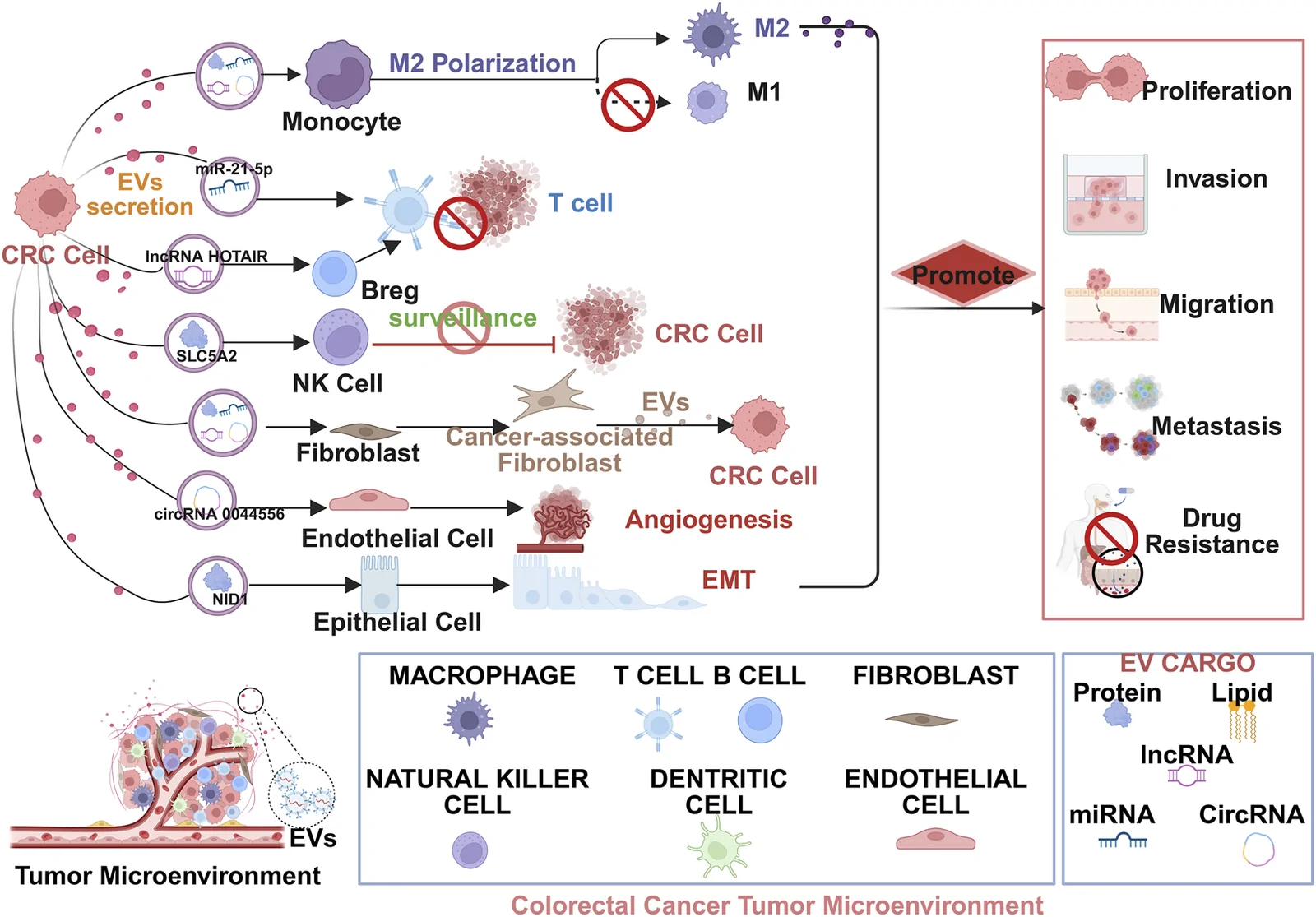
EVs-Mediated Crosstalk Among Components of the CRC Tumor Microenvironment. CRC-derived EVs directly enhance the proliferation, migration, and drug resistance of adjacent tumor cells, promote the establishment of PMNs, and induce the malignant transformation of normal epithelial cells. In addition, CRC-derived EVs promote the polarization of monocytes toward the M2 phenotype, suppress the function of CD8^+^ T cells and B cells, enable escape from NK cell-mediated immune surveillance, and promote the transformation of fibroblasts into CAFs. (This figure was created with Biorender.com).

### 2.1 The effect of EVs on the TME in CRC

The various components of the TME collectively contribute to tumor initiation and progression. In addition to tumor cells, the TME comprises immune cells (including macrophages, T cells, B cells, dendritic cells, and neutrophils), fibroblasts, mesenchymal stem cells, and other stromal populations (Yang et al., 2025). As key mediators of intercellular communication, EVs indirectly reshape the immunosuppressive TME, thereby driving CRC progression and malignancy.

#### 2.1.1 EVs promote M2 polarization of macrophages

Tumor-associated macrophages (TAMs) represent one of the most abundant immune populations within the CRC tumor microenvironment, and their polarization status critically influences CRC progression (Nicolini et al., 2024). EVs can polarize macrophages into two distinct phenotypes: classically activated, pro-inflammatory, antitumor M1-like macrophages, and alternatively activated, anti-inflammatory, protumor M2-like macrophages (Xia et al., 2023). CRC-derived EVs drive the polarization of TAMs from the M0 toward the M2 phenotype, thereby facilitating the progression of the malignant phenotype (Hui et al., 2023). We next focus on the RNA and protein cargo of EVs that drives M2 polarization.

Numerous studies have focused on EV-encapsulated microRNAs (miRNAs) as key cargo molecules that regulate M2 polarization. EV-derived miR-21-5p and miR-200a have been identified as key signaling molecules that mediate the crosstalk between CRC cells and macrophages. Specifically, by modulating the phosphatase and tensin homolog/protein kinase B (PTEN/AKT) and suppressors of cytokine signaling 1/signal transducer of activation 1 (SOCS1/STAT1) pathways, they synergistically induce M2-like polarization and programmed cell death ligand 1 (PD-L1) upregulation in macrophages, thereby suppressing CD8^+^ T-cell activity and promoting CRC growth (Yin et al., 2022). Highly metastatic CRC cells release miR-106a-5p-loaded EVs, which promote M2 polarization of macrophages by inhibiting SOCS6 and activating the janus kinase 2/signal transducer of activation 3 (JAK2/STAT3) pathway, thereby facilitating CRC liver metastasis (Liang et al., 2024). Zhao et al. reported, through bioinformatics analysis, that miR-92a-3p is upregulated in plasma EVs from CRC patients. Mechanistically, miR-92a-3p activates the mitogen-activated protein kinase/extracellular signal-regulated kinase (MAPK/ERK) pathway to promote M0-to-M2 macrophage polarization, thereby enhancing CRC cell migration and angiogenesis (Zhao et al., 2025). Overexpression of miR-203a-3p is closely associated with poor prognosis and liver metastasis in CRC patients. EV-derived miR-203a-3p is upregulated in the plasma of CRC patients and in the highly metastatic CRC cell line HCT116. This miRNA induces M2 polarization of macrophages by regulating PTEN and activating the phosphatidyl-inositol-3-kinase (PI3K)/AKT signaling pathway. M2-polarized macrophages secrete C-X-C motif ligand 12 (CXCL12), which promotes cancer metastasis via the CXCL12/C-X-C receptor 4 (CXCR4)/nuclear factor-kappaB (NF-κB) axis and establishes a pre-metastatic niche in colorectal cancer liver metastasis (CRLM), thereby driving CRC liver metastasis (Pei et al., 2023). In a related study, Shi et al. reported that plasma EVs from CRC patients exhibit high expression of miR-372-5p. Mechanistically, miR-372-5p—which is highly enriched in HCT116-derived EVs—is phagocytosed by macrophages and promotes their M2 polarization through regulation of the PTEN/AKT pathway. These M2 macrophages subsequently secrete CXCL12, which in turn activates the WNT/β-catenin pathway, thereby enhancing EMT, stemness, and metastatic potential in CRC cells (Shi et al., 2023).

In addition to miRNAs, circular RNAs (circRNAs) have also been shown to regulate macrophage polarization by sponging miRNAs. Sun et al. reported that circPOLQ is significantly upregulated in CRC tumor tissues compared with normal tissues and is associated with poor prognosis. Using *in vitro* experiments, they further demonstrated that CRC cell-derived EVs enriched in circPOLQ promote M2 macrophage polarization by targeting miR-379-3p and activating the interleukin-10 (IL-10)/STAT3 axis (Sun Z. et al., 2024). Tang et al. reported that circVCP is highly enriched in the plasma and cells of CRC patients. Mechanistically, CRC cell-derived EVs carrying circVCP promote proliferation, migration, and invasion by regulating the miR-9-5p/neuropilin-1 (NRP1) axis, and concomitantly induce M2 polarization while suppressing M1 polarization (Tang et al., 2023). Similarly, circUBQLN1 is significantly elevated in CRC-derived EVs. These EVs are internalized by TAMs and, via the miR-34c-5p/CSF1R signaling axis, induce their transition toward an M2-like phenotype (Wang D. et al., 2025). Notably, long non-coding RNAs (lncRNAs) also contribute to macrophage polarization. LINC01615, which is highly expressed in CRC tissues, is transported from CRC cells to TAMs via EVs, where it mediates the RNA-binding motif protein X-linked- Enhancer of zeste homolog 2 (RBMX-EZH2) axis to promote M2 polarization and thereby facilitate CRC progression (Li S. et al., 2025a).

Beyond RNAs, EV-encapsulated proteins also modulate macrophage function. 3-Hydroxy-3-methylglutaryl coenzyme A synthase 1 (HMGCS1) catalyzes the conversion of acetyl-CoA to HMG-CoA, thereby facilitating cholesterol biosynthesis. Yang et al. reported that HMGCS1 is highly expressed in CRC cells; HMGCS1 promotes the conversion of acetyl-coenzyme A to HMG-CoA, thereby enabling these cells to synthesize cholesterol and release cholesterol-containing EVs, which in turn promote M2 polarization of macrophages (Yang et al., 2025a). CRC cell-derived EVs containing HIF-2α bind to the CXCL12 promoter, promoting CXCL12 secretion by peritumoral fibroblasts, which in turn induces M2 macrophage accumulation and tumor cell invasion (Wang et al., 2025a). In an *in vitro* study, Ding et al. reported that EVs from SW620 and HCT-15 CRC cells carry the lncRNA BANCR, which activates the Ras homolog family member A/Rho-associated coiled-coil containing protein kinase (RhoA/ROCK) pathway by recruiting insulin-like growth factor 2 binding protein 2 (IGF2BP2), thereby promoting M2 macrophage polarization. In turn, these M2-polarized macrophages enhance CRC cell proliferation and invasion (Ding et al., 2024). Additionally, CRC-derived EVs mediate the transfer of ETS proto-oncogene 2 (ETS2) to macrophages, where it targets pre-mRNA processing factor 4 (PRPF4) to promote M2 polarization and CD8^+^ T-cell apoptosis, thereby leading to immunosuppression (Hou et al., 2026). CRC cell-derived EVs contain CD133, which induces TAM polarization toward the M2 phenotype. These M2-like TAMs subsequently secrete IL-6, which activates the STAT3 pathway in CRC cells and promotes tumor proliferation and EMT (Kim et al., 2024). E2F transcription factor 7 (E2F7) is significantly upregulated in CRC tissues and positively correlates with CD163^+^ macrophage infiltration. CRC-derived EVs transfer E2F7 to macrophages, where it induces an immunosuppressive M2-like phenotype through binding to the structural maintenance of chromosome 4 (SMC4)(Gao et al., 2026). CT-26 CRC cell-derived EVs highly express heat shock protein 90 beta family member 1 (HSP90B1). Upon macrophage uptake, these EVs induce the M2 phenotype while simultaneously reducing CD8^+^ T-cell survival, thereby promoting the formation of immunosuppressive PMNs that facilitate liver metastasis (Li S. et al., 2025b).

Collectively, these studies are predominantly based on *in vitro* findings and await validation in more robust *in vivo* models and large patient cohorts to confirm their prevalence and functional relevance, which would better inform clinical translation.

#### 2.1.2 The role of EVs in immunosuppression

Tumor cells evade immune surveillance through various mechanisms. CRC-derived EVs, in particular, regulate immune cell function through multiple pathways, most notably by suppressing CD8^+^ T-cell activity and proliferation, thereby driving cancer progression (Abu et al., 2020). Tumor-derived EVs suppress the antitumor activity of CD8^+^ T cells through multiple mechanisms, representing a key route of immune evasion. CRC cell-derived EVs carrying miR-21-5p and miR-200a synergistically induce M2-like macrophage polarization and PD-L1 upregulation through regulation of the PTEN/AKT and SOCS1/STAT1 pathways, thereby suppressing CD8^+^ T-cell activity and promoting tumor growth (Yin et al., 2022). Wu et al. reported that miR-372-5p is upregulated in CRC tissues, serum, cell lines, and their corresponding EVs. Macrophages internalize EVs secreted by the HCT116 CRC cell line, which modulates PD-L1 expression and ultimately establishes an immunosuppressive microenvironment that dampens T-cell activation. When macrophages treated with miR-372-5p are co-cultured with T cells, they modulate PD-L1 expression via the PTEN/AKT/NF-κB pathway, resulting in reduced CD3^+^CD8^+^ T-cell activity (Wu J. et al., 2024).

In addition to the well-established role of CD8^+^ T cells in tumor immunosuppression, CD4^+^ T cells have also garnered considerable attention. In CRC patients, elevated PD-L1 levels on sEVs correlate with more advanced disease stages. Analysis using an *in vitro* co-culture system and flow cytometry revealed that CRC-derived EVs transport PD-L1 to macrophages and impair CD4^+^ T-cell function (Wang et al., 2025b).

EVs also induce immunosuppression by modulating B cells. sEVs secreted by the murine CRC cell line MC38 induce B cells to acquire an immunosuppressive phenotype and suppress their proliferation and activity. Moreover, MC38-derived EVs induce B cells to acquire regulatory B-cell (Breg) characteristics, with upregulation of IL-10 and Transforming growth factor beta (TGF-β). Concurrently, B cells treated with MC38-derived EVs attenuate CD8^+^ T-cell functional activity (Zhang et al., 2025). Similarly, CRC-derived EVs carrying the lncRNA HOTAIR induce B cells to polarize into PD-L1-expressing Bregs. These PD-L1+ Bregs subsequently suppress CD8^+^ T-cell activity, thereby reinforcing immunosuppression (Xie et al., 2023).

Evasion of immune surveillance by natural killer (NK) cells represents another critical factor in immunosuppression. Sodium-glucose cotransporter 2 (SLC5A2) exerts an oncogenic function in CRC cells. Xiao et al. reported that SLC5A2 regulates membrane potential and calcium influx and, via EVs, enhances the secretion of major histocompatibility class I (MHC-I) -associated major histocompatibility complex-class I chain related proteins A and B (MICA/B), thereby enabling CRC cells to evade NK cell surveillance (Xiao et al., 2026).

Collectively, these findings indicate that EVs carry a diverse array of molecules that regulate immune cells through multiple mechanisms; however, the synergistic effects of these EV-mediated pathways in reshaping the immune microenvironment warrant further investigation.

#### 2.1.3 The bidirectional effects of EVs on CRC fibroblasts

In CRC, EVs induce the transformation of fibroblasts into CAFs, thereby driving tumor progression. CRC cell-derived EVs promote the conversion of fibroblasts into CAFs, whereas CAF-derived EVs, in turn, act back on CRC cells, establishing a bidirectional crosstalk loop (Rahmati et al., 2024).

##### 2.1.3.1 CRC-derived EVs induce the transformation of fibroblasts into CAFs

Zhang et al. reported that HSPC111 levels in serum EVs, primary tumors, and CAFs were significantly higher in CRC patients with liver metastases than in those without metastases. HSPC111 modulates lipid metabolism in CAFs by phosphorylating ATP-citrate lyase (ACLY), thereby upregulating acetyl-coenzyme A (acetyl-CoA) levels. Accumulated acetyl-CoA subsequently promotes CXCL5 expression and secretion by enhancing H3K27 acetylation in CAFs. HSPC111 in CRC-derived sEVs promotes PMN formation and CRC liver metastasis (Zhang et al., 2022).

Zhou et al. further revealed a bidirectional crosstalk mechanism between CRC cells and CAFs. MiR-99a-5p carried by CRC-derived EV (EV-miR-99a) specifically targets NLR family pyrin domain containing 2 (NLRP2) mRNA in fibroblasts and activates the pro-inflammatory NF-κB pathway, thereby converting normal fibroblasts into CAFs. EV-miR-99a-activated CAFs enhance CRC cell migration by secreting C-C motif chemokine ligand 7 (CCL7). Moreover, TGF-β1 secreted by CAFs upregulates miR-99a expression in CRC cells in an NF-κB-dependent manner, thereby establishing a miR-99a/TGF-β1 regulatory loop (Zhou et al., 2026).

The PMN is a critical driver of CRLM, and hepatic stellate cells (HSCs) contribute to CRLM by differentiating into CAFs. CRC-derived EVs carrying integrin αvβ3 activate the mitogen-activated protein kinase (MEK)-ERK signaling pathway via the vitronectin-integrin αvβ3-focal adhesion kinase (FAK) axis, thereby promoting HSC activation. Once transformed into CAFs, HSCs promote PMN formation through EMT, angiogenesis, and inflammatory mediators, thereby facilitating CRLM (Yu et al., 2026).

Research in this area has primarily focused on EV-encapsulated RNAs and proteins, with limited in-depth exploration of EV-associated DNA. Wang et al. reported that PIK3CA-mutated DNA in CRC-derived EVs is delivered to recipient fibroblasts, where it undergoes transcription and translation. Ultimately, through interaction with the endogenous P85 regulatory subunit of phosphatidylinositol 3-kinase (PI3K), this DNA-derived product activates fibroblasts and transforms them into CAFs. These CAFs promote tumor cell migration *in vitro* by secreting elevated IL-6 levels and facilitate lung metastasis *in vivo* (Wang et al., 2022). Similarly, C-X-C motif chemokine receptor 7 (CXCR7) is an atypical chemokine receptor. In CRC cells overexpressing CXCR7, the levels of miR-146a-5p and miR-155-5p increase coordinately in both the cells and their EVs. These CRC-derived miR-146a-5p and miR-155-5p are taken up by CAFs via EVs and, by targeting SOCS1 and zinc finger and BTB domain-containing 2 (ZBTB2), promote CAF activation through the JAK2-STAT3/NF-κB pathway. In turn, these activated CAFs further enhance the invasive capacity of CRC cells (Wang R. et al., 2025).

##### 2.1.3.2 EVs derived from CAFs promote the malignant phenotype of CRC cells

High mobility group box 3 (HMGB3), a member of the HMGB family, promotes CRC progression through regulation of the WNT/β-catenin pathway. Yang et al. demonstrated that circ N4BP2L2 in CAF-derived EVs promotes CRC cell proliferation, migration, and angiogenesis while suppressing apoptosis through regulation of the miR-664b-3p/HMGB3/WNT/β-catenin pathway. Using a nude mouse model, they suppressed subcutaneous tumor growth and liver metastasis by knocking down circ N4BP2L2 (Yang et al., 2022).

Extensive evidence indicates that CAF-secreted EVs carry miRNAs that modulate CRC-related signaling pathways, thereby promoting the malignant phenotype of CRC cells. For instance, CAF-derived EVs deliver miR-135b-5p to colorectal adenocarcinoma (COAD) cells, where they downregulate forkhead box O1 (FOXO1) and promote proliferation, migration, and angiogenesis in human umbilical vein endothelial cells (HUVECs) (Dai et al., 2022). CAF-derived EVs induce nuclear translocation of the NF-κB p65 transcription factor in SW480 CRC cells, leading to increased IL-6 production and subsequently promoting CRC progression (Ghofrani-Shahpar et al., 2024). CAF-derived EVs carrying miR-181b-3p modulate CRC cells by regulating sorting nexin 2 (SNX2) expression. Further *in vitro* and *in vivo* experiments collectively confirmed that miR-181b-3p drives CRC pathogenesis by upregulating SNX2 expression (Jiang et al., 2023). miR-522-3p in CAF-derived sEVs promotes CRC cell migration, invasion, and angiogenesis by downregulating bone morphogenetic protein 5 (BMP5), suggesting a potential therapeutic target for CRC (Zhang et al., 2024). CAF-derived EVs carrying miR-6765-3p promote the malignant phenotype of CRC by stimulating aerobic glycolysis through regulation of the G Protein Subunit Gamma 7/mammalian target of rapamycin (GNG7/mTOR) pathway (Xi et al., 2026).

In addition to the aforementioned miRNA-centric studies, a limited number of investigations have addressed circRNAs and lncRNAs. For example, circ_0067557 and circN4BP2L2—both upregulated in CAF-derived EVs—promote CRC cell proliferation, migration, and chemoresistance (Qu et al., 2022; Yang et al., 2024). CAF-derived EVs enriched in circTAX1BP1 drive CRLM(Tan et al., 2025) by promoting VIR-like m6A methyltransferase associated (VIRMA) lactylation and specific protein 1 (SP1)/TGF-β signaling, thereby forming a positive feedback loop via ITGA11^+^ myCAFs. LINC00355 in CAF-derived EVs promotes EMT and chemoresistance in CRC through regulation of the miR-34b-5p/CRKL axis (Hu et al., 2024).

Ferroptosis—an iron-dependent form of cell death characterized by iron accumulation and reactive oxygen species (ROS) generation—also plays a critical role in CRC progression. Ren et al. demonstrated that methyltransferase-like 3 (METTL3) is enriched in CAFs and packaged into EVs, where it promotes CRC cell proliferation and metastasis while suppressing ferroptosis by inducing ACSL3 m6A modification (Ren et al., 2024). In a related study, Guan et al. reported that circLDLR is packaged into CAF-derived EVs and transferred to CRC cells, where it protects polypeptide N-acetylgalactosaminyltransferase 14 (GALNT14) from zinc and ring finger 2 (ZNRF2)-mediated ubiquitination and degradation, thereby stabilizing the protein. This stabilization promotes GALNT14 membrane localization and ultimately suppresses ferroptosis in CRC cells, thereby enhancing their proliferation and metastasis (Guan et al., 2026).

Collectively, these findings on CAF-CRC crosstalk provide a robust theoretical foundation for elucidating CRC pathogenesis. Building on this foundation, we are better positioned to develop more personalized therapeutic strategies for CRC.

### 2.2 The role of EVs in CRC metastasis

Metastasis is a major cause of mortality in CRC, with liver metastasis representing the most frequent site of dissemination (Zhou J. et al., 2024). Accumulating evidence indicates that CRC-derived EVs promote EMT, angiogenesis, and PMN formation (Wu Y. et al., 2024).

#### 2.2.1 EVs regulate endothelial cells to promote angiogenesis

Tumor angiogenesis is critical for tumor growth, as it supplies the nutrients and oxygen required for tumor progression. Angiogenesis is also essential for rapid tumor growth and dissemination (Rahmati et al., 2024). A study demonstrated that EVs derived from the HT-29 CRC cell line promote tumor growth and angiogenesis in a murine CRC model. Using *in vitro* experiments, the authors found that HT-29-derived EVs are internalized by HUVECs and modulate vascular endothelial growth factor-A (VEGFA) expression in endothelial cells via the JAK/STAT3 pathway, thereby promoting angiogenesis in CRC tumors. These findings provide a theoretical basis for EV-targeted anti-tumor strategies in CRC and potentially other malignancies (Long et al., 2024). Additionally, Hu et al. reported that hsa_circ_0044556 functions as an oncogene in CRC and is strongly associated with angiogenesis. CRC cell-derived EVs carrying hsa_circ_0044556 promote angiogenesis in HUVECs by recruiting eukaryotic translation initiation factor 4A3 (EIF4A3). Their *in vitro* experiments further demonstrated that hsa_circ_0044556-loaded EVs promote angiogenesis by activating the Smad2/3 signaling pathway and accelerate tumor growth and angiogenesis *in vivo*. These findings offer new insights into anti-angiogenic targeted therapies for CRC (Hu et al., 2023).

#### 2.2.2 EVs regulate the malignant phenotype of epithelial cells

In tumor progression, EMT promotes tumor metastasis. EMT is characterized by downregulation of epithelial markers (E-cadherin, β-catenin, and claudins) and upregulation of mesenchymal markers (vimentin, N-cadherin, and fibronectin) (Quan et al., 2024, Wang W. et al., 2025). HSCs undergo phenotypic transition from a quiescent to a highly proliferative and motile state, thereby influencing CRC cell behavior through extracellular matrix (ECM) remodeling (Chen et al., 2025).

Pucci et al. isolated EVs from the culture supernatants of SW480 and SW620 CRC cells, as well as from plasma of CRC patients and healthy controls, and used them to treat human hepatic THLE-2 cells. They found that CRC-derived EVs carrying TGF-β1 impair the morphological and functional properties of THLE-2 cells by triggering TGF-β1/SMAD-dependent EMT, thereby promoting EMT in hepatic cells and facilitating the formation of a fibrotic microenvironment that favors CRC metastasis (Pucci et al., 2023). Chen et al. reported that nidogen 1 (NID1) is present in EVs from metastatic CRC, and that EV-NID1 promotes EMT in CRC cells by modulating EMT-related genes. Concurrently, EV-NID1 activates HSCs, which in turn stimulate neutrophil infiltration and, through secretion of IL-11, induce the formation of neutrophil extracellular traps (NETs) in the liver metastatic microenvironment. This process ultimately reshapes the TME and promotes the establishment of a niche conducive to CRLM(Chen et al., 2025).

Yang et al. demonstrated that CRC-derived EVs deliver fibrinogen gamma (FGG), thereby promoting angiogenesis in HUVECs. Furthermore, FGG downregulates VE-cadherin and E-cadherin while upregulating N-cadherin and vimentin in CRC cells, thereby increasing endothelial permeability and promoting EMT. *In vivo* studies further showed that FGG downregulates VE-cadherin in CRC tissues while upregulating CD31 in liver tissues, ultimately leading to an increased number of liver metastatic nodules in a murine CRC liver metastasis model (Yang et al., 2025b).

5-fluorouracil (5-FU) is a standard chemotherapeutic agent for CRC. However, acquired resistance to 5-FU remains a major clinical challenge. Zhang et al. demonstrated that circ-0023919 promotes the migration and invasion of 5-FU-resistant CRC cells both *in vitro* and *in vivo*, while also enhancing their chemoresistance to 5-FU. Mechanistically, circ-0023919 acts as a molecular sponge for miR-197-5p, thereby upregulating intercellular adhesion molecule-5 (ICAM-5) expression. By promoting EMT, circ-0023919 enhances CRC cell resistance to 5-FU. Thus, circ-0023919 may represent a potential therapeutic target for CRC (Zhang et al., 2026).

Collectively, these findings provide new insights into the mechanisms of CRC liver metastasis and offer potential avenues for therapeutic intervention.

### 2.3 EVs mediate PMN formation

Prior to metastasis, tumor cells establish a PMN that favors metastatic colonization (Li T. et al., 2025). The primary tumor microenvironment is thought to facilitate metastasis to secondary sites, a process conceptually described by the classic “seed and soil” hypothesis, wherein tumor cells represent the “seeds” and the PMN constitutes the “soil.” When the “soil” environment is more conducive to growth, it becomes more favorable for the “seeds” to metastasize to that organ (Kaplan et al., 2005).

Zhou et al. reported that circ-0034880 is highly expressed in plasma EVs from CRC patients and is closely associated with CRLM. Tumor-associated EVs (TEVs) enriched in circ-0034880 home to the liver and activate SPP1ʰ^ig^ʰCD206^+^ pro-tumor macrophages, thereby promoting PMN formation and facilitating liver metastasis (Zhou J. et al., 2024). In a related study, Li et al. reported that plasma EV miR-188-3p expression was significantly higher in CRC patients with liver metastases than in non-metastatic patients. CRC-derived EVs carrying miR-188-3p promote liver metastasis by activating the AKT/mTOR pathway via pleckstrin homology domain leucine-rich repeat protein phosphatase 2 (PHLPP2) targeting, thereby stimulating HSCs to form PMNs(Li Y. et al., 2025).

Collectively, these findings underscore the critical role of PMN establishment in CRC liver metastasis, although the underlying molecular mechanisms warrant further elucidation.

## 3 The application of EVs in CRC treatment

Although the therapeutic armamentarium for CRC is diverse—including surgery, chemotherapy, radiotherapy, targeted therapy, and immunotherapy—treatment resistance has remained a major obstacle to successful clinical outcomes. EVs can potentiate immunotherapeutic responses, and both mesenchymal stem cell (MSC)-derived EVs and engineered EV platforms have emerged as promising strategies for CRC-targeted therapy (Figure 2).

**FIGURE 2:**
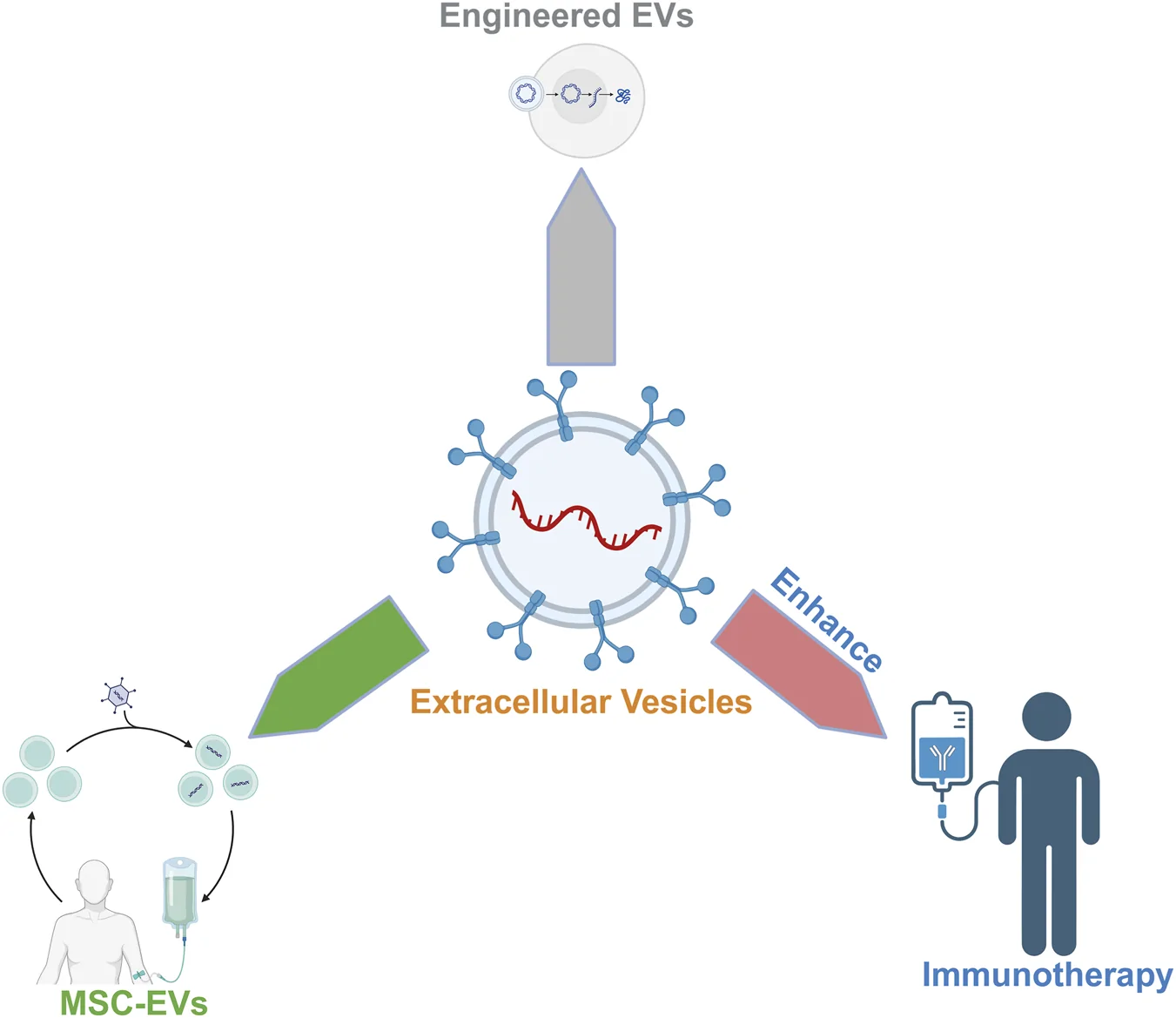
EVs as therapeutic vehicles in CRC. EVs have shown tremendous potential in CRC therapy, including immunoenhancement, stem cell-derived EV therapy, and engineered EV-based therapeutic applications. (This figure was created with Biorender. com).

### 3.1 EVs as an adjuvant to immunotherapy

Dietary probiotics have been shown to enhance the efficacy of immunotherapy in CRC. Lu et al. isolated EVs from *Lactobacillus* rhamnosus GG (designated LGG-EV). LGG-EV demonstrated superior gastrointestinal stability in both *in vitro* and *in vivo* settings. Oral administration of LGG-EV synergistically enhanced the efficacy of anti-PD-1 immunotherapy in CRC. Mechanistically, LGG-EV modulated gut immunity by increasing the CD8^+^/CD4^+^ T-cell ratio in mesenteric lymph nodes and elevating the proportions of MHC-II^+^ dendritic cells, CD4^+^ T cells, and CD8^+^ T cells within tumor tissues. Furthermore, significant changes were observed in the levels of serum metabolites associated with the microbiome and antitumor effects, including uridine—whose levels increased following combined anti-PD-1 and LGG-EV therapy (Lu et al., 2023). In a related study, Li et al. proposed an innovative therapeutic platform combining selenium nanoparticle-loaded EVs, interleukin-32, and engineered *Escherichia coli* Nissle 1917. This platform significantly enhanced CD8^+^ T-cell proliferation and activity and delayed CRC progression in humanized murine models. Both *in vitro* and *in vivo* experiments confirmed that this platform exhibits favorable biocompatibility and antioxidant properties, and enhances CRC immunotherapy efficacy while alleviating immune tolerance (Li Y. et al., 2025).

Additionally, bacteria-derived EVs also serve as both vaccines and therapeutic tools for CRC. One study demonstrated that synthetic bacterial vesicles (SyBV) loaded with a stimulator of interferon Genes (STING) agonist enhance antitumor effects; these modified vesicles synergistically activate dendritic cells and promote interferon-β production. *In vivo* experiments further demonstrated that these EVs synergistically inhibit melanoma and CRC growth by enhancing T-cell infiltration into tumors (Lötvall et al., 2025). These naturally derived EVs exhibit favorable tissue compatibility and represent a promising avenue for CRC therapy.

Dendritic cell (DC)-based antitumor immunotherapy has demonstrated significant therapeutic potential. TEVs provide a rich source of tumor-associated antigens. However, unmodified TEVs contain high levels of immunosuppressive molecules that inhibit biological processes involved in antigen processing and presentation to naive T lymphocytes. Rossowska et al. used lentiviruses to engineer the CRC cell line MC38, resulting in TEVs that overexpress IL-18 and/or shRNAs targeting IL-10 or TGF-β1. These engineered EVs potently stimulated DCs, thereby enhancing their therapeutic efficacy and significantly inhibiting CRC growth. This work provides a foundation for next-generation DC-based vaccines (Rossowska et al., 2026). MiR-155, one of the most extensively studied miRNAs, plays a crucial role in DC maturation and IL-12 production. Asadirad et al. investigated the antitumor effects of DCs pretreated with miR-155-rich EVs in a CT-26-induced CRC model in BALB/c mice. They first introduced miR-155 into TEVs via electroporation, then treated bone marrow-derived DCs with miR-155-rich TEVs, and subsequently evaluated the antitumor properties of these primed DCs in a BALB/c CRC model. The results showed that primed DCs significantly elevated serum IL-12 and interferon-gamma (IFN-γ) levels, accelerated the differentiation, proliferation, and cytotoxic activity of Th cells and cytotoxic T lymphocytes (CTLs,) and simultaneously increased their infiltration into the TME while reducing regulatory T-cell (Treg) numbers. This mechanism contributes to tumor growth control and improved survival. Therefore, DC-based immunotherapy using miR-155-enriched TEVs may represent a promising strategy for inducing antitumor immune responses, suppressing tumor growth, and prolonging survival in CRC-bearing mice. Nevertheless, further in-depth studies are warranted to validate the clinical utility of this approach in humans and its applicability to other tumor types (Asadirad et al., 2022).

### 3.2 EVs derived from mesenchymal stem cells

Stem cell therapy has been widely explored not only for cancer but also for various other diseases. Compared with traditional stem cell therapies, mesenchymal stem cell (MSC)-derived EVs offer several advantages: (i) superior biocompatibility, which mitigates the risk of adverse differentiation associated with live cell transplantation; (ii) versatile administration routes, higher delivery efficiency, and the ability to cross the blood-brain barrier. These properties position MSC-EVs as a promising therapeutic modality for CRC(Wang C. et al., 2025).

Antisense oligonucleotides (ASOs) represent a novel class of targeted therapeutic agents for cancer. Yu et al. utilized human umbilical cord mesenchymal stem cell (hUC-MSC)-derived EVs as delivery vehicles for an anti-miR-146b-5p ASO (PMO-146b). By covalently conjugating PMO-146b to the anchoring peptide CP05(P)—which recognizes the MSC-EV surface marker CD63—they assembled the complex onto the EV surface, thereby establishing a novel therapeutic platform. Following treatment with ePPMO-146b, they assessed cell proliferation, uptake, migration, and EMT vitro. They further evaluated its *in vivo* antitumor efficacy and biodistribution in a murine xenograft model. The results showed that the platform was taken up by SW620 cells and effectively inhibited their proliferation and migration. Following systemic administration, this platform is enriched in tumor tissue from a mouse xenograft model of colorectal cancer (Yu et al., 2024).

Most studies have focused on the therapeutic effects mediated by miRNAs delivered via MSC-EVs into CRC cells. For example, MSC-EV-mediated delivery of miR-34a-5p inhibits CRC development and progression by regulating c-MYC binding to DNA methyltransferase 3a (DNMT3a) and by epigenetically modulating PTEN (Zhao J. et al., 2022). Jahangiri et al. treated CRC cells with MSC-EVs and found that these EVs, enriched in miR-100 and miR-143, downregulated mTOR, Cyclin D1, K-RAS, and HK2 while upregulating p-27. MSC-EVs inhibit CRC cell proliferation, migration, invasion, and metastasis via the miR-100/mTOR/miR-143 axis, while also inducing apoptosis (Jahangiri et al., 2022). Ye et al. reported that engineered human MSCs (hMSCs) efficiently load miR-3614-5p into their secreted EVs. These engineered EVs effectively inhibit CRC progression by downregulating IL-7Rα and suppressing the JAK2/STAT3 pathway (Ye et al., 2026). Jiang et al. reported that MSC-EV-derived miR-125b-5p significantly suppressed the expansion, proliferation, and immunosuppressive function of Tregs *in vitro*. Furthermore, they observed marked tumor growth retardation in mice treated with these engineered MSC-EVs. Notably, while anti-PD-1 monotherapy achieved a cure rate of approximately 30% in a murine CRC model, combination with miR-125b-5p-loaded EVs significantly enhanced therapeutic efficacy, inducing tumor regression in approximately 80% of treated mice—a 2- to 3-fold improvement over anti-PD-1 alone. This enhanced efficacy was associated with reduced tumor-infiltrating Tregs and increased CTLs (Jiang et al., 2025).

Collectively, these findings underscore the considerable therapeutic potential of MSC-EVs. However, their clinical translation remains constrained by several limitations, including the lack of standardized production protocols, the potential presence of endogenous carcinogenic factors, and insufficient long-term safety data from clinical follow-up.

### 3.3 Engineered EVs

Engineered EVs loaded with anticancer molecules can directly modulate the proliferation, migration, and invasive phenotypes of CRC cells, thereby exerting therapeutic effects. Although research in this area remains at an early stage, the studies discussed below provide a theoretical foundation for EV-based therapeutic strategies. Plant-derived EVs have emerged as novel players in cancer diagnosis and therapy. Several studies have engineered natural plant-derived EVs to address the challenges of oral chemotherapy for CRC. Oral chemotherapy for CRC is hampered by poor tumor selectivity and microenvironment-driven drug resistance. To overcome these limitations, Lv et al. designed a cyclic transferrin (Tf)-binding peptide (cp) with high Tf affinity and gastrointestinal stability, and engineered ginger-derived EVs (cp-GEVs) to enable *in situ* Tf-mediated “sandwich-style” targeted delivery. By recruiting endogenous Tf, cp-GEVs establish a Tf-mediated bridging interface that selectively interacts with intestinal epithelial cells and tumor cells overexpressing Tf receptors. This enables efficient transcellular transport across the intestinal epithelium, tumor-selective accumulation, and deep tumor penetration following oral administration. When loaded with irinotecan (CPT-11), CPT@cp-GEVs significantly enhanced intracellular drug delivery efficiency and reprogrammed immunosuppressive M2-like tumor-associated macrophages toward a pro-inflammatory phenotype, thereby disrupting the drug-resistant microenvironment enriched in cancer stem cells. This study presents a conceptually unique material design paradigm that combines endogenous ligand recruitment with immune microenvironment reprogramming (Lv et al., 2026). In a related study, Hao et al. demonstrated that an oral capsule containing capecitabine (EVOC)—constructed from lemon-derived EVs—can transiently open the intestinal epithelial barrier through mechanical stress, thereby significantly improving drug delivery efficiency. This oral formulation substantially enhances chemotherapeutic efficacy in CRC (Hao et al., 2026). Broccoli contains a range of bioactive compounds with established health benefits. However, efforts to overcome 5-fluorouracil (5-FU) resistance in CRC have thus far met with limited success. Studies have shown that broccoli-derived EVs (BEVs) isolated from broccoli juice are effectively taken up by HT-29 CRC cells. Co-administration of BEVs and 5-FU significantly inhibits HT-29 CRC cell proliferation and migration, and effectively arrests the cell cycle. BEVs reverse HT-29 cell resistance to 5-FU by suppressing the aberrant activation of the PI3K/AKT/mTOR pathway. This approach thus represents an effective strategy for sensitizing CRC cells to 5-FU chemotherapy (Cao et al., 2024).

In addition to plant-derived EVs, milk-derived and microbe-derived EVs have also been engineered to exert therapeutic effects or enhance chemosensitivity. Multimodal image-guided photothermal therapy (PTT) offers advantages including minimal side effects and robust therapeutic efficacy. There is an urgent need to develop PTT nanocarriers with high drug-loading efficiency and surface-modification capability. Goat milk-derived EVs (GMVs) have emerged as an ideal PTT nanocarrier platform owing to their anti-inflammatory properties, tumor retention capacity, high yield, and favorable biosafety profile. Jing et al. analyzed the key genes, biological processes, and signaling pathways involved in indocyanine green (ICG)-mediated PTT and N3-GMV@ICG-mediated PTT. This nanoprobe triggers antitumor immune and inflammatory responses, thereby enhancing PTT efficacy; moreover, owing to the anti-inflammatory properties of GMVs, it simultaneously alleviates PTT-induced inflammatory responses (Jing et al., 2023). Microbe-derived EVs (MEVs) provide a biocompatible, naturally derived platform for drug delivery. Lee et al. encapsulated doxorubicin in *Lactobacillus* plantarum-derived EVs. *In vitro* experiments revealed that these MEVs maintained structural stability under physiological conditions, and their endocytosis involved clathrin- and dynamin-mediated pathways (Lee S. et al., 2026).

KRAS is a common oncogenic driver, yet its targeted therapy remains challenging. Sun et al. developed engineered EVs that enable *in vivo* self-assembly of small EVs (sEVs) encapsulating KRAS-specific siRNA, thereby achieving broad-spectrum targeted therapy against KRAS mutants. Following hepatic uptake, these sEVs encapsulate the siRNA and display the CRC-targeting peptide TCP-1 on their surface, thereby enabling tumor-specific delivery. This approach achieved highly efficient tumor-targeted therapy, significantly suppressed tumor growth, and led to complete tumor regression in some cases (Sun Y. et al., 2026). Oxaliplatin (OXA) is a first-line chemotherapeutic agent for CRC, and sensitivity to resistance directly affects patient prognosis. However, the precise underlying mechanisms remain incompletely understood. Li et al. used electroporation to construct engineered EVs carrying siMDM2 and validated their therapeutic value in enhancing OXA-based chemotherapy. This effect was mediated by inhibition of the murine double minute 2/new MD-related candidate gene/ROS (MDM2/NDUFB8/ROS) axis in patient-derived xenograft (PDX) models as well as in murine models of liver and lung metastasis, providing new insights and experimental evidence for overcoming platinum resistance in CRC (Li A. et al., 2025).

CRC is characterized by epidermal growth factor receptor (EGFR) overexpression, which is often associated with poor prognosis and chemoresistance. Li et al. developed EGFR-targeted EVs (EGFR-tEVs) by transiently engineering donor cells to express the GE11 peptide, aiming to enhance the precision of doxorubicin (Dox) delivery. *In vitro* experiments demonstrated that EGFR-tEV-Dox exhibited higher cellular uptake in EGFR-overexpressing HCT-116 cells, thereby activating the p53-Bax-mediated poly (ADP-ribose) (PAR) polymerase 1 (PARP1) apoptotic pathway. These findings indicate that EGFR-tEV-Dox enhances functional drug delivery to malignant tissues while minimizing apoptotic effects in normal cells (Lee C. M. et al., 2026).

Engineered EVs offer improved targeting and delivery efficiency for CRC; however, their clinical translation faces persistent challenges, including batch-to-batch reproducibility, uncertain immunogenicity, scalability, and cost. Moreover, no large-scale preclinical trials or dose-finding studies have yet been conducted to establish safety profiles for clinical application.

## 4 Challenges in clinical translation: heterogeneity, standardization, and safety issues

Currently, no EVs for the treatment of CRC have been approved for marketing. The main barriers to this are threefold (summarized in Table 1):

1. Lack of a gold standard for isolation and purification. Currently, multiple methods are available for EV purification—including differential ultracentrifugation, size-exclusion chromatography, and immunomagnetic bead separation—each differing in specificity and yield, leading to substantial batch-to-batch variability in EV purity and quantity. As recommended by the Minimal Information for Studies of Extracellular Vesicles (MISEV) guidelines (2018 and 2023), the appropriate isolation method should be selected based on the requirements of downstream applications (Théry et al., 2018; Welsh et al., 2024). Furthermore, large-scale, standardized EV production remains a major challenge, and inter-batch consistency and stability are difficult to guarantee. Mature quality control standards for key EV parameters—including drug-loading efficiency, *in vivo* stability, and release kinetics—are still lacking (Lu et al., 2025). Moreover, EVs are inherently heterogeneous with respect to size, cellular origin, cargo composition, and biological function. In terms of diagnosis, the broad spectrum of EVs poses significant challenges for diagnostic analysis; single-cell-derived EV analysis is required to improve diagnostic accuracy, but this technology is costly and technically demanding. In terms of treatment, EVs from different sources exhibit significant differences in cargo-loading efficiency (Zhang et al., 2025a).
2. Therapeutic challenges. Directly targeting the pathways involved in EV biogenesis or secretion may disrupt normal intercellular communication, posing unknown systemic risks to immune homeostasis and tissue regeneration (Maurya et al., 2026). A meta-analysis by Delen et al. concluded that the incidence of serious adverse events (liver dysfunction, fever, vomiting, acute asthma) associated with EVs therapy was 0.7%, and the probability of experiencing an adverse event was 4.4% (Van Delen et al., 2024). Furthermore, engineering EVs remains technically demanding, with a low success rate. In addition, establishing a safe and effective therapeutic dose remains difficult (Fan et al., 2022).
3. Translational and regulatory gaps. Most EV-related clinical applications remain concentrated in diagnostics (NCT04523389, NCT07754513, NCT07319104, NCT04852653, NCT06955767), whereas only a limited number of therapeutic studies have progressed to the patient recruitment phase. Furthermore, ethical considerations surrounding the clinical use of EVs—for example, those derived from human umbilical cord MSCs—remain complex and require careful evaluation (Yuan et al., 2023).

**TABLE 1:** Barriers to the clinical translation of extracellular vesicles.

Obstacle level	Core challenge	Specific manifestations
Technical bottlenecks	Lack of standardization	Inconsistent isolation methods lead to incomparable results
​	Difficulties in production and quality control	Challenges in scalable production; significant batch-to-batch variability
​	Intrinsic heterogeneity	Bulk analysis masks signals from key subpopulations
Clinical application barriers	Diagnostic value to Be validated	Insufficient biomarker validation; unclear added value over existing tools
​	Safety concerns in therapy	Risk of off-target effects and potential disruption of normal physiological functions
​	Delivery and quantification hurdles	Unclear pharmacokinetics; poor controllability of drug loading and targeting
Translation pathway gaps	Scarce clinical evidence	Mostly early-phase studies; lack of large-scale prospective trials
​	Empty regulatory framework	Ambiguous product classification; no established precedents for approval pathways

## 5 Conclusion

EVs play a multifaceted regulatory role in CRC pathogenesis by carrying diverse bioactive molecules, including miRNAs, lncRNAs, circRNAs, and DNA. On the one hand, EVs remodel the TME by promoting macrophage M2 polarization, suppressing T- and B-cell activity and proliferation, and enabling tumor cells to evade NK cell surveillance, thereby establishing an immunosuppressive microenvironment. Moreover, EVs interact with tumor-associated fibroblasts to further promote tumor progression. On the other hand, EVs directly drive CRC metastasis by stimulating endothelial angiogenesis, inducing EMT, and promoting PMN formation. These mechanisms indicate that EVs serve as crucial communication mediators linking tumor cells, immune cells, and stromal cells in CRC pathogenesis.

Based on the role of EVs in the pathogenesis of CRC, numerous studies have demonstrated that EVs can be used for the treatment of CRC. These therapeutic approaches include: using EVs to enhance the efficacy of immunotherapy; utilizing EVs derived from mesenchymal stem cells to deliver therapeutic molecules; and engineering EVs—including plant-derived and microbial-derived EVs—to improve their targeting ability and stability, thereby providing new directions for CRC treatment.

Currently, the challenges in clinical translation primarily center on the following areas: the lack of a gold standard for isolation and purification; safety concerns regarding *in vivo* therapy; and unresolved ethical issues. In future research, we can develop standardized, scalable isolation and purification technologies; advance the design of therapeutic clinical trials; and refine relevant regulations regarding the ethics of treatment.

The potential clinical implications of these findings are primarily reflected in three areas. In terms of diagnosis, the various biomarkers carried by EVs can be used for the early detection of CRC, prognostic assessment, and monitoring of treatment efficacy; most current clinical studies are focused on these areas. In terms of treatment, EVs can serve both as therapeutic targets—inhibiting CRC progression by blocking their oncogenic signaling—and as therapeutic carriers, delivering immunomodulatory molecules, chemotherapeutic agents, or gene therapy tools to enhance the efficacy of immunotherapy and overcome drug resistance. In terms of translation, EVs derived from mesenchymal stem cells and engineered EVs offer new therapeutic modalities for CRC. Overall, EVs research offers an important direction for the precise diagnosis and personalized treatment of CRC; however, its clinical translation still requires multidisciplinary collaboration and systematic validation.

## Glossary

EVs: extracellular vesicles;
CRC: colorectal cancer
TME: tumor microenvironment
CAFs: cancer-associated fibroblasts
EMT: epithelial-mesenchymal transition
EMT: epithelial-mesenchymal transition
PMN: pre-metastatic niche
TAMs: tumor-associated macrophages
miRNAs: microRNAs
circRNAs: circular RNAs
PTEN: phosphatase and tensin homolog
AKT: protein kinase B
SOCS1: suppressors of cytokine signaling 1
STAT: signal transducer of activation
PD-L1: programmed cell death ligand 1
JAK2: janus kinase 2
MAPK: Mitogen-activated protein kinase
ERK: Extracellular signal-regulated kinase
PI3K: phosphatidyl-inositol-3-kinase
CXCL12: C-X-C motif ligand 12
CXCR4: C-X-C receptor 4
NF-κB: nuclear factor-kappaB
LncRNAs: long non-coding RNAs
CRLM: colorectal cancer liver metastasis
IL-10: interleukin-10
NRP1: neuropilin-1
RBMX: RNA-binding motif protein X-linked
EZH2: Enhancer of zeste homolog 2
HMGCS1: 3-Hydroxy-3-methylglutaryl coenzyme A synthase 1
IGF2BP2: insulin-like growth factor 2 binding protein 2
RhoA: Ras homolog family member A
Rock: Rho-associated coiled-coil containing protein kinase
ETS2: ETS proto-oncogene 2
MVs: microvesicles
PRPF4: pre-mRNA processing factor 4
E2F7: E2F transcription factor 7
SMC4: the structural maintenance of chromosome 4
HSP90B1: heat shock protein 90 beta family member 1
TGFβ: transforming growth factor beta
NK: natural killer
Breg: regulatory B Cell
SLC5A2: Sodium-glucose cotransporter 2
MHC-I: Major histocompatibility class I
MICA/B: major histocompatibility complex-class I chain related proteins A and B
ACLY: ATP-citrate lyase
CoA: coenzyme A
NLRP2: NLR family pyrin domain containing 2
CCL7: C-C motif chemokine ligand 7
HSCs: hepatic stellate cells
FAK: focal adhesion kinase
MEK: Mitogen-activated protein kinase
PI3K: phosphatidylinositol 3-kinase
CXCR7: C-X-C motif chemokine receptor 7
ZBTB2: zinc finger and BTB domain-containing 2
HMGB3: High mobility group box 3
COAD: colorectal adenocarcinoma
FOXO1: Forkhead box proteins O1
HUVECs: human umbilical vein endothelial cells
SNX2: sorting nexin 2
BMP5: bone morphogenetic protein 5
GNG7: G Protein Subunit Gamma 7
mTOR: mammalian target of rapamycin
VIRMA: VIR-like m6A methyltransferase associated
SP1: Specific protein 1;
ROS: reactive oxygen species
METTL3: methyltransferase-like 3
GALNT14: polypeptide N-acetylgalactosaminyltransferase 14
ZNRF2: zinc and ring finger 2
VEGFA: vascular endothelial growth factor-A
EIF4A3: eukaryotic translation initiation factor 4A3
ECM: extracellular matrix
NETs: neutrophil extracellular traps
NID1: nidogen 1
FGG: fibrinogen gamma
5-FU: 5-Fluorouracil
ICAM-5: intercellular adhesion molecule-5
TEVs: tumor-derived EVs
PHLPP2: pleckstrin homology domain leucine-rich repeat protein phosphatase 2
MSC: mesenchymal stem cell
LGG-EV: Lactobacillus rhamnosus GG
PD-1: programmed death-1
SyBV: synthetic bacterial vesicles
STING: stimulator of interferon Genes
DC: Dendritic cell
IFN-γ: interferon-gamma
CTLs: cytotoxic T lymphocytes
Tregs: regulatory T-cell
MSC: mesenchymal stem cell
ASOs: Antisense oligonucleotides
hUC-MSC: human umbilical cord mesenchymal stem cell
PMO-146b: anti-miR-146b-5p ASO
DNMT3a: DNA methyltransferase 3a
hMSCs: human MSCs
Tf: transferrin
cp: cyclic transferrin-binding peptide
CPT-11: irinotecan
EVOC: capecitabine
5-FU: 5-fluorouracil
BEVs: broccoli-derived EVs
PTT: Multimodal image-guided photothermal therapy
GMVs: Goat milk-derived EVs
ICG: indocyanine green
MEVs: Microbe-derived EVs
sEVs: small EVs
OXA: Oxaliplatin
PDX: patient-derived xenograft
MDM2: Murine double minute 2
NDUFB8: New MD-related candidate gene
EGFR: epidermal growth factor receptor
EGFR-tEVs: EGFR-targeted EVs
DOX: doxorubicin
PARP1: poly (ADP-ribose) (PAR) polymerase 1
MISEV: Minimal Information for Studies of Extracellular Vesicles
